# *KSN* heterozygosity is associated with continuous flowering of *Rosa rugosa* Purple branch

**DOI:** 10.1038/s41438-021-00464-8

**Published:** 2021-02-01

**Authors:** Mengjuan Bai, Jinyi Liu, Chunguo Fan, Yeqing Chen, Hui Chen, Jun Lu, Jingjing Sun, Guogui Ning, Changquan Wang

**Affiliations:** 1 College of Horticulture, Nanjing Agricultural University, Nanjing, 210095 China; 2 College of Horticulture and Forestry Sciences, Huazhong Agricultural University, Wuhan, 430070 China

**Keywords:** Flowering, Histone post-translational modifications

## Abstract

Rose (*Rosa* spp.) plants flower via two contrasting methods: once flowering (OF) and continuous flowering (CF). Purple branch is a rare continuously flowering variety of *Rosa rugosa* that is extensively cultivated in China. However, the genetic basis of its CF behavior is unknown. We demonstrated that Purple branch is heterozygous for the *TFL1* homolog *KSN*. One *KSN* allele with a 9 kb Copia insertion was found to be identical to that from continuously flowering *Rosa chinensis* Old blush. The other allele was found to be a functional wild-type allele. The overall expression of *KSN* was closely linked to the floral transition, and it was significantly repressed in continuously flowering Purple branch compared with OF Plena. The promoter region of the normal *KSN* allele was hypermethylated, and histone methylation at H3H4, H3K9, and H3K27 of the *KSN* gene locus was modified in continuously flowering Purple branch. Silencing of the DNA methyltransferase genes *MET1* and *CMT3* and the histone methyltransferase gene *SUVR5* in Purple branch led to enhanced *KSN* expression, but silencing of the histone demethylase gene *JMJ12* suppressed *KSN* expression. Therefore, the CF habit of Purple branch may be due to reduced expression of *KSN* caused by the halved dose and may be associated with epigenetic modifications together with retrotransposon insertions along the chromosome. Our study revealed a novel mechanism underlying the CF behavior of rose plants.

## Introduction

*Rosa rugosa* Thunb., a member of the Rosaceae family, which is indigenous to Eastern Asia, was introduced into Europe and North America in the middle of the nineteenth century^1,2^. In addition to producing fragrant flowers, this popular and economically valuable ornamental species has medicinal properties. *R. rugosa* flowers are widely used in traditional and folk medicine in China, Japan, and Korea due to the presence of secondary metabolites that exert pharmacological activities^3^. Essential oil from petals, known as “liquid gold”, is a raw material used in perfumes, cosmetics, aromatherapy, spices, and the nutrition industry^4^. Unlike modern roses (*Rosa hybrida*), which flower continuously throughout the year, most *R. rugosa* varieties flower once a year during the spring. Efforts to produce flowers year-round rely on screening and breeding new continuously flowering *R. rugosa* varieties.

Flowering, which involves the transition from vegetative growth to reproductive development, is controlled by external environmental cues and endogenous signals through six genetic pathways in *Arabidopsis*: the photoperiod, vernalization, autonomous, gibberellin, age, and temperature pathways^5^. Rose plants have three flowering methods: an once flowering (OF) period in the spring; continuous flowering (CF) during favorable growth seasons; and occasional reblooming^6,7^.

Rose *KSN*, a homolog of *TFL1* of *Arabidopsis thaliana*, acts as a flowering repressor to control OF/CF. In OF rose cultivars, the expression of *KSN* is repressed in the winter and early spring, so the plants bloom only in late spring. After blooming, *KSN* expression is activated and represses further flower formation in the summer^6,8,9^. Continuously flowering *Rosa chinensis* Old blush contains a 9 kb Copia retrotransposon insertion in the second intron of *KSN*, and this insertion blocks *KSN* expression, enabling continuous flowering^9^. Old blush is hybridized with European rose plants to generate modern rose plants, in which the recurrent flowering trait has presumably been transferred into the modern rose plants^6^. Therefore, most continuously flowering rose cultivars are expected to harbor this mutated allele of *KSN* from Old blush. However, the presence of a null allele has been suggested in the haploid Old blush genome sequence and possibly contributes to its CF behavior^10^, and some continuously flowering rose plants have no Copia insertion in their *KSN* gene^11,12^; thus, the mutation of *KSN* may not be the only reason for the rose CF trait.

In Arabidopsis, *TFL1* maintains the indeterminate growth of the inflorescence meristem by inhibiting *AP1* and *LFY* expression^13,14^. In *tfl1* mutants, indeterminate meristems are rapidly converted into determinate meristems, which produce terminal flowers^13,15,16^. Functional characterization of *FvTFL1* has confirmed its role as a floral repressor that causes OF: mutations in this gene lead to CF in strawberry^9,17^. In perennial woody plant species, silencing the *TFL1* homolog shortens the juvenile period and causes continuous flowering. For example, silencing *MdTFL1* in apple (*Malus domestica*) reduces the juvenile period from 5 years to several months^18,19^, whereas silencing *PcTFL1* in pear (*Pyrus communis* L.) accelerates flowering by 1–8 months^20^. In *Populus* spp., silencing of the *TFL1* orthologs *PopCEN1*/*PopCEN2* hastens the first onset of flowering, maintains the indeterminate growth of axillary meristems and accelerates bud dormancy release upon chilling^21^. In perennial *Arabis alpine* plants, *AaTFL1* regulates the duration of age-dependent vernalization required for *AaLFY* expression and sets a threshold for flowering^22^. Therefore, *TFL1* (*KSN*) is functionally conserved as a flowering repressor.

**Fig. 1:**
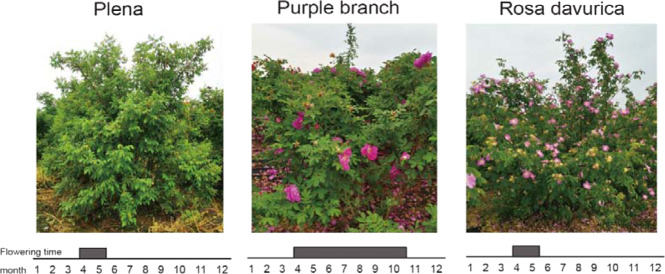
Images of *R. rugosa* Plena, *R. rugosa* Purple branch and *R. davurica*. *R. rugosa* Plena and *R. davurica* are OF species blooming from April to May. *R. rugosa* Purple branch is a continuously flowering variety that flowers from April to October. The flowering time is represented by the gray boxes

Purple branch is a rare continuously flowering variety of *R. rugosa* that is cultivated widely in China. *R. rugosa* Purple branch provides raw materials for the production of essential oils, jams, teas, pies, beverages, and other derivatives, integrating the primary, secondary and tertiary industries. To date, the origin of Purple branch remains unclear, but it is generally considered to have derived from an interspecific cross between *R. rugosa* Plena and *Rosa davurica*^23,24^. Intriguingly, its presumptive parents are OF rose species (Fig. 1); thus, its CF behavior remains unexplained. Elucidating the molecular mechanism of the CF trait in Purple branch will provide a theoretical basis for breeding new varieties with different flowering phenotypes.

In this paper, we demonstrate that the continuously flowering *R. rugosa* Purple branch is heterozygous for *KSN*: it contains one normal transcribed wild-type allele and another transcriptionally blocked allele with a 9-kb Copia insertion, identical to that in *R. chinensis* Old blush. We show that the CF behavior of Purple branch is associated with reduced *KSN* expression. This reduction is due to the halved dose of the wild-type *KSN* allele and is linked to promoter hypermethylation and histone modification at the *KSN* locus. We thus present a novel mechanism for the production of the CF habit of rose plants.

## Materials and methods

### Plant materials and growth conditions

*R. rugosa* Purple branch, *R. rugosa* Plena, *R. davurica* and *R. chinensis* Old blush were grown in the rose resource nursery of Nanjing Agricultural University (Nanjing, China) under natural conditions.

*Arabidopsis thaliana* wild-type (Columbia), *tfl1-14* mutant and transgenic lines were grown in a grown chamber under controlled conditions (22 °C, 40% relative humidity, and 180 μmol m^−2^ s^−1^ of photosynthetically active radiation) under long-day conditions (16 h of light/8 h of darkness).

### Gene cloning and expression analysis

Genomic DNA from Purple branch, Plena, *R. davurica* and Old blush leaves was isolated by the CTAB method^25^. The different fragments of the *KSN* gene were isolated by PCR using the primers listed in Table S1. For expression analysis, shoot apices of field-grown Purple branch, Plena and *R. davurica* were collected on 29 March, 11 April and 30 September 2019, flash frozen in liquid nitrogen, and then stored at −80 °C. Total RNA was extracted using a Vazyme FastPure Plant Total RNA Isolation Kit (Polysaccharides & Polyphenolics rich, Vazyme Biotech, Nanjing, China). One microgram of high-quality total RNA was reverse transcribed using TransScript One-Step gDNA Removal and cDNA Synthesis SuperMix (Transgen Biotech, Beijing, China) according to the manufacturers’ instructions. The cDNA was amplified via RT-PCR, and quantitative reverse-transcription PCR (qRT-PCR) of *KSN* and *FT* was carried out with ABI QuantStudio 5 instrument (ABI Life Technologies, Carlsbad, USA) using the primers listed in Table S1; R*cGAPDH* was used as an internal reference^26^. Each experiment was conducted for three biological replicates, with three technical repeats each.

### Methylation-specific PCR (MSP)

Methylation of the *KSN* promoter was studied using MSP. Less than 500 ng of genomic DNA was treated with sodium bisulfite using an EZ DNA Methylation Kit (Zymo Research, Irvine, USA). The treated DNA was subsequently used as a template for PCR. Approximately 1 kb of the *KSN* promoter region was amplified using two pairs of primers (Table S1) that were designed via Methyl Primer Express v1.0. The total volume of the PCR was 25 μl, and PCR was performed using TaKaRa EpiTaq HS (for bisulfite-treated DNA) according to the following protocol: 35 cycles of 98 °C for 10 s, 55–60 °C for 30 s, and 72 °C for 30 s, followed by 72 °C for 10 min. The resulting PCR fragments were ligated into a pMD-18-T vector (TaKaRa, Dalian, China), which was then transformed into *Escherichia coli* (Trans-T1) (Transgen Biotech, Beijing, China), and 10 clones per sample were sequenced using a bioanalyzer. Each experiment was performed for three biological repeats. The methylation percentages of cytosine (C) in CG, CHH and CHG (H=A, C or T) were analyzed using online software (http://katahdin.mssm.edu/kismeth/revpage.pl), and the running parameters were as follows: minimum fraction of positive matches, 0.8; minimum fraction of length, 0.5.

### Chromatin immunoprecipitation PCR (ChIP-PCR)

ChIP-PCR was performed as described previously^27^, with slight modifications. Shoot apices of field-grown Purple branch and Plena were collected in September 2020 and ground in liquid nitrogen in M1 buffer (10 mM phosphate buffer [pH 7.0], 0.1 M NaCl, 10 mM mercaptoethanol, 1 M hexylene glycol, 1× protease inhibitor cocktail [Roche], 5% PVP, and 1 mM PMSF). The suspension was filtered through four layers of Miracloth, after which the filtrate was centrifuged at 12,000 rpm for 10 min. The pelleted chromatin was washed thrice with M2 buffer (M1 buffer plus 10 mM MgCl_2_ and 0.5% Triton X-100) and once with M3 buffer (10 mM phosphate buffer [pH 7.0], 0.1 M NaCl, 10 mM mercaptoethanol, 1× protease inhibitor cocktail [Roche], and 1 mM PMSF). The chromatin was resuspended in nuclear lysis buffer and sonicated to generate DNA fragments of approximately 500 bp. The lysate was precleared by incubation together with 50 µl of protein-A agarose beads/salmon sperm DNA (Millipore, Billerica, USA) for 1 h. It was then incubated together with IgG, anti-H3K4me3, anti-H3K9me3, and anti-H3K27me2 antibodies (Abcam, Cambridge, UK) overnight. The bound DNA fragments were recovered and purified using columns from a plasmid extraction kit (Qiagen, Hilden, Germany) according to the manufacturer’s instructions. Quantitative real-time PCR was subsequently performed using the bound and input DNA as templates in conjunction with the primers listed in Table S1.

### Transient transformation system

The coding regions of *MET1*, *CMT3*, *JMJ12*, and *SUVR5* of *R. rugosa* Purple branch were cloned using the primers listed in Table S1 and then sequenced. The resulting sequences were used for phylogenetic analysis with *Arabidopsis thaliana* to validate their reliabilities and then submitted to the NCBI database.

To silence specific genes using RNAi, the conserved fragments of *MET1* (MW012566), *CMT3* (MW012565), *JMJ12* (MW012568) and *SUVR5* (MW012567) were amplified from cDNA of Purple branch using specific primers (Supplemental Table S1). The 300–400 bp amplicons were then subcloned into a pENTR-D-TOPO vector (Invitrogen, Carlsbad, USA). A pFAST-R03 binary vector (http://www.psb.ugent.be/) was used in the subsequent LR recombination to generate RNAi plasmids.

To analyze the RNAi effects, young shoots of Purple branch were transiently transformed as previously described^28^. Briefly, young shoots were placed into a 50-mL Agrobacterium solution carrying the gene fragments of interest and vacuum infiltrated for 5 min, and an Agrobacterium solution carrying the empty vector served as a control. After being released from the vacuum, the shoots were washed with deionized water, and the leaves were collected for RNA extraction and q-PCR analysis after 3 days.

### Statistical analyses

Statistical analysis of the data was performed via SPSS 17 statistical software. Two groups of data were compared using Kolmogorov–Smirnov (KS) tests (*P < 0.05; **P < 0.01). Multiple groups of data were compared using the Kruskal–Wallis (KW) test, with *P* < 0.05 considered significant.

## Results

### Purple branch contains two KSN alleles

**Fig. 2:**
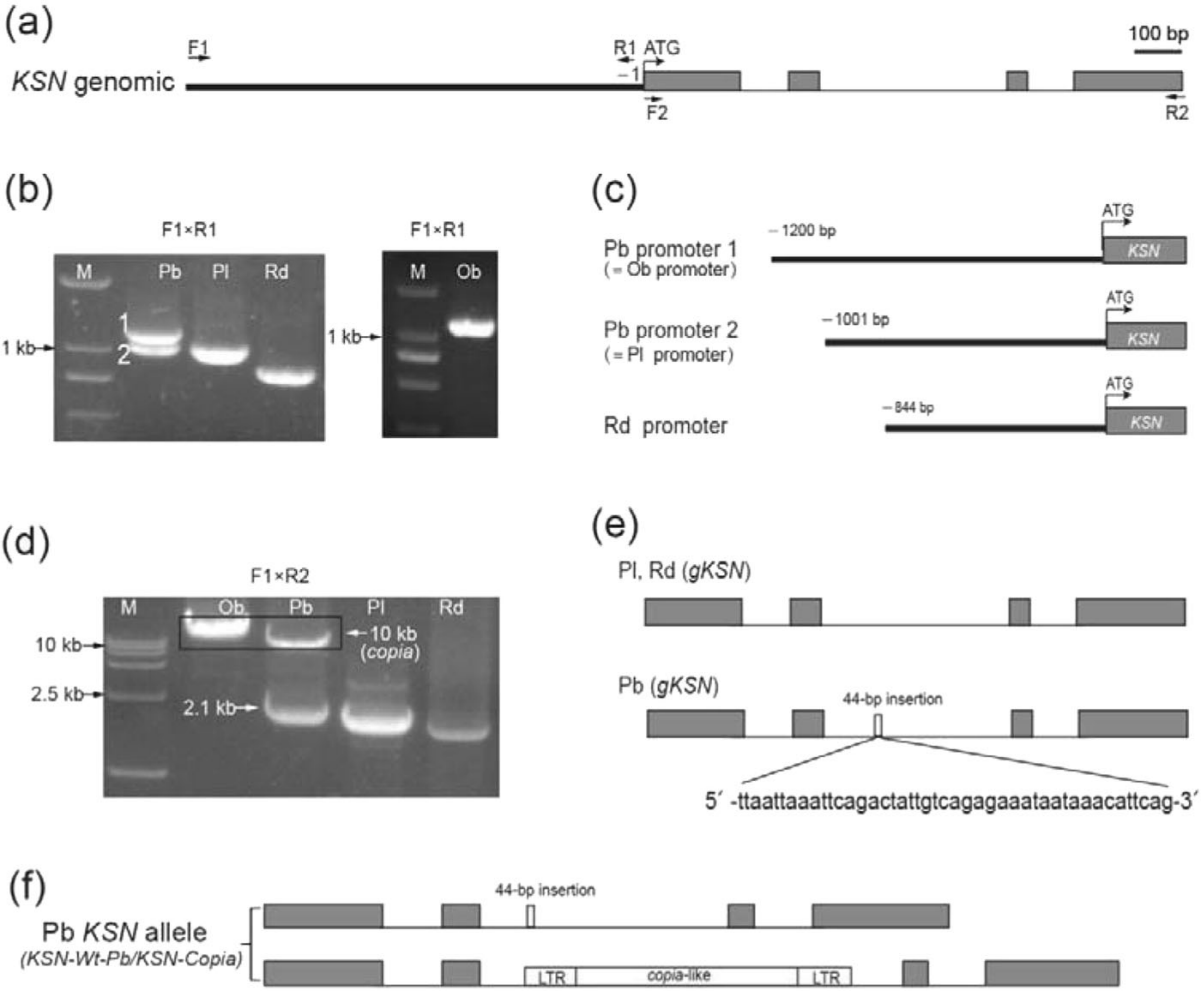
Heterozygous *KSN-Copia*/*KSN-Wt-Pb* in continuously flowering Purple branch. **a** Schematic representation of the *KSN* gene in Rosaceae. The promoter is represented by a thick black line, exons are represented by gray boxes, introns are represented by thin black lines, and primers are indicated with arrows. **b** PCR products separated on an agarose gel by electrophoresis using primers F1/R1 in Purple branch (Pb), Plena (Pl), *R. davurica* (Rd) and *R. chinensis* Old blush (Ob). Only one band is amplified in OF Pl and Rd, whereas there are two bands (1 and 2) in CF Pb. **c** Sequence alignment indicating that Pb promoter 1 is identical to the *KSN* promoter in Ob and that Pb promoter 2 is identical to the *KSN* promoter in Pl. **d** PCR amplification of *KSN* using primers F1/R2 in Pb, Pl, Rd and Ob. **e** Sequence alignment of the 2.1 kb product in Pb: the second intron contains a 44 bp insertion compared with the second intron of Pl and Rd. **f** Schematic representation of the heterozygous *KSN-Copia*/*KSN-Wt-Pb* in CF Pb

We cloned the *KSN* promoter and coding region from continuously flowering Purple branch, OF Plena, and *R. davurica* using the same primers that were previously used to amplify this region in *R*. *chinensis* Old blush^29^. We used the primer pair F1/R1 to amplify the promoter fragment and the primer pair F1/R2 to clone the full-length *KSN* gene, including both the promoter and coding areas (Fig. 2a). Based on the results of electrophoresis analysis of the PCR products on agarose gels, we obtained two bands from the Purple branch, with primers F1/R1 or F1/R2, and only one band of *KSN* from Plena and *R. davurica* (Fig. 2b, d).

Subsequent sequence alignment showed that the sequence of one of the *KSN* alleles from Purple branch was almost the same as that from Plena, except for a few SNPs and a 44 bp insertion in the second intron. The other allele was similar to that from CF *R*. *chinensis* Old blush and contained a Copia retrotransposon insertion in the second intron (Figs. 2c, e and S1). These results indicate that the diploid Purple branch is heterozygous for *KSN* (*KSN-Copia*/*KSN-Wt-Pb*) (Fig. 2f). Because neither allele was similar to that of *R. davurica*, our work contradicts the notion that Purple branch was derived from an interspecific cross between *R. rugosa* Plena and *R. davurica*.

### *KSN* expression is lower in Purple branch than in Plena and *R. davurica*

**Fig. 3:**
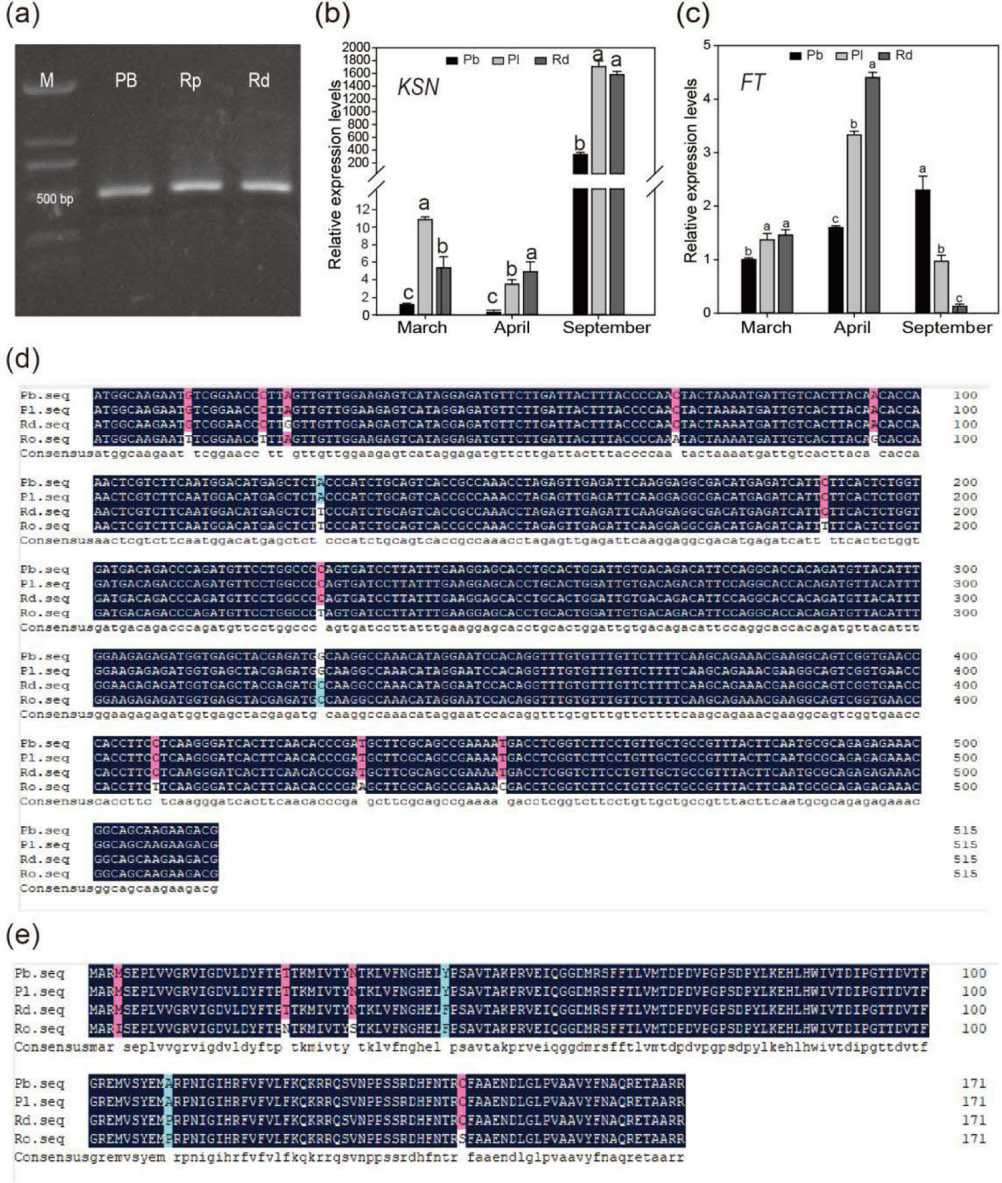
Expression analysis of *KSN* from Purple branch, Plena and *R. davurica*. **a** PCR amplification of *KSN* using primers F2/R2 from cDNA of Purple branch (Pb), Plena (Pl) and *R. davurica* (Rd). **b**, **c** Expression levels of *KSN* and *FT* from Pb, Pl, and Rd in March, April, and September. **d**, **e** Show the nucleotide sequence and amino acid sequence alignments of *KSN* genes from Pb, Pl, Rd, and *R. wichurana* (Ro). The letters above the bars indicate significant differences, as determined by the KW test (*P* < 0.05)

Because one of the *KSN* alleles contains a 9 kb Copia insertion in the second intron in Purple branch, we tested whether the other allele contains a 44 bp insertion in the second intron that was transcribed normally. We cloned the coding regions of *KSN* from the cDNA of Purple branch, Plena, and *R. davurica*. We obtained a single band from the three different genotypes and sequenced the amplicons (Fig. 3a). Purple branch was identical to Plena (Fig. 3d, e) and contained a few bases that were different from those of *R. davurica* and *R*. *chinensis* Old blush. Thus, continuously flowering Purple branch carrying a wild-type *KSN* allele can be transcribed normally without interference by the 44 bp insertion.

To further compare the expression levels of *KSN* among Purple branch, Plena, and *R. davurica* and determine the link between *KSN* and flowering modes, we conducted qRT-PCR on samples collected in March (before blooming), April (during blooming), and September (continuously flowering Purple branch blooms, but OF *R. rugosa* Plena and *R. davurica* do not bloom) from all three genotypes. *KSN* expression was inhibited before flowering, maintained at relatively low levels during flowering, but obviously increased after flowering in September not only in OF *R. rugosa* Plena and *R. davurica* but also in continuously flowering Purple branch (Fig. 3b). However, the *KSN* expression in continuously flowering Purple branch being dramatically lower than that in OF Plena and *R. davurica* at all three time points may be caused by halved doses, leading to a CF habit. We also analyzed *FT* expression at the same time points. The expression levels of *FT* in Purple branch, Plena, and *R. davurica* were all higher in April than in March, and the levels still increased in September in continuously flowering Purple branch but declined significantly in OF *R. rugosa* Plena and *R. davurica* (Fig. 3c). Therefore, *KSN* expression was negatively associated with the flowering transition in the spring, and the flowering of Purple branch in autumn may be due to the integrative effects of both *KSN* and *FT*.

### *KSN* from Purple branch rescues the early-flowering *tfl1* mutant phenotype

To determine the functional conservation of the *KSN* transcript from Purple branch, we expressed this gene under the constitutive 35S promoter in the Arabidopsis *tfl1* mutant background. We then examined the flowering phenotype and gene expression levels in the transgenic plants.

**Fig. 4:**
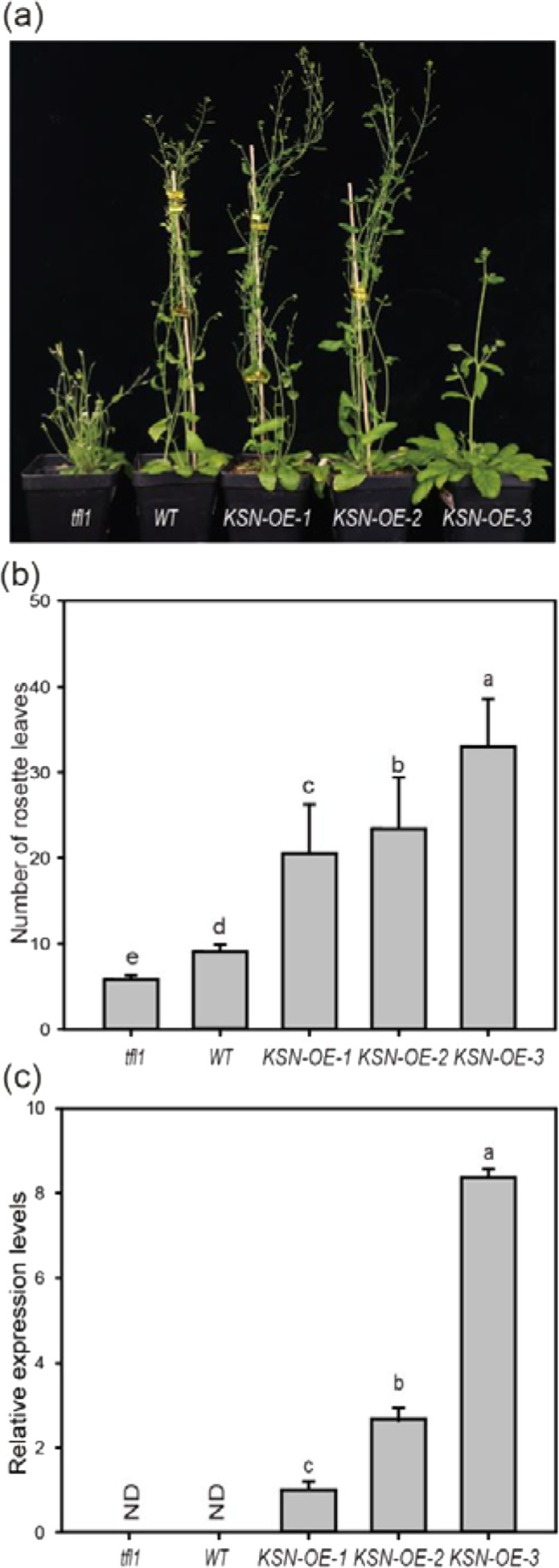
Ectopic expression of KSN from continuously flowering Purple branch rescues the early flowering phenotype of the Arabidopsis *tfl1* mutant. **a** Phenotype of Arabidopsis wild type (WT); *tfl1-14* mutants; and the *KSN* heterologous expression lines *KSN-OE-1*, *KSN-OE-2*, and *KSN-OE-3*. **b** Rosette leaves of the above genotypes at flowering. **c** Expression levels of *KSN* in the above genotypes. The error bars indicate the standard deviations (*n* = 3). The different letters above the bars indicate significant differences, as determined by the KW test (*P* < 0.05)

The heterologous expression of *KSN* from Purple branch rescued the early flowering and determinate growth of the inflorescence of *tfl1* mutants (Fig. 4a, b). The *tfl1-14* mutant produced 5.8 rosette leaves in contrast to the 9 rosette leaves produced by wild-type Arabidopsis at flowering. The flowering of the *KSN-OE-1* and *KSN-OE-2* lines was delayed to occurring when there were more than 20 rosette leaves. This delay was associated with increased *KSN* mRNA levels. Seedlings of the *KSN-OE-3* line, with relatively high *KSN* expression levels, were more delayed and produced more than 30 rosette leaves before flowering (Fig. 4b, c).

In addition, some transgenic seedlings produced leaf-like floral organs that generated no seeds. These results corroborated those from studies of overexpression of *TFL1* from *Vitis vinifera*, *Lotus japonicus* and *Rosa wichurana* in Arabidopsis^30–32^. Thus, the *KSN* of Purple branch is the functional homolog of *TFL1* in Arabidopsis and other species and acts as a floral repressor.

### The *KSN* promoter in Purple branch is hypermethylated

**Fig. 5:**
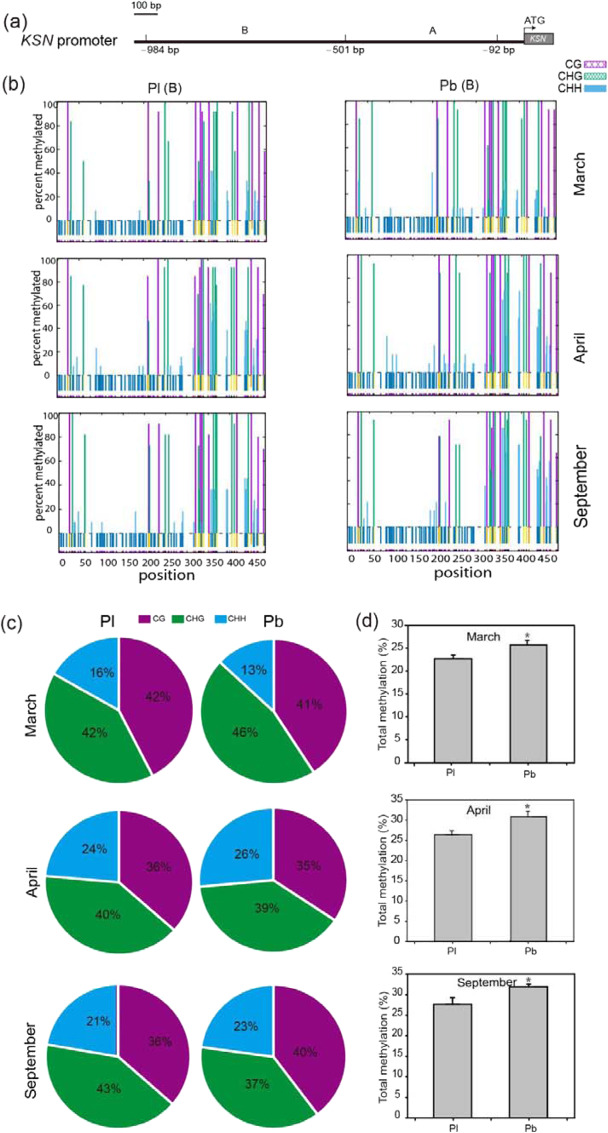
Region B methylation status of the *KSN* promoter in Purple branch and Plena. **a** Schematic representation of fragment B (–984 to –501 bp) and fragment A (–501 to –92 bp) of the *KSN* promoter. **b** The methylation status of CG/CHG/CHH sites in region B of *KSN* promoters from Purple branch (Pb) and Plena (Pl) in March, April, and September. The colored lines above the X-axis show the percentage of methylation at individual cytosine sites. The short bars at the bottom of the graph show the positions of the cytosines. **c** The pie chart shows the percentage of region B with three types of methylation at different stages in Pb and Pl. **d** Comparisons of the total methylation percentage of region B in Pb and Pl according to Kismeth software (http://katahdin.mssm.edu/kismeth/revpage.pl). The error bars indicate the standard deviations (*n* = 3). The asterisks above the bars indicate significant differences, as determined by KS tests (*P < 0.05)

We hypothesized that the heterozygosity-induced dose decrease may not be the only reason for the inhibition of *KSN*. The 9 kb insertion may alter the chromatin structure and induce epigenetic suppression or activation. Using MSP, we tested the methylation status of CpG islands in the promoter of the normally transcribed *KSN* allele in Purple branch. The B fragment (–984 to –502 bp) rather than the A fragment (–501 to –92 bp) was identified as the CpG island for DNA methylation (Fig. 5a, b). The total percentages of the three types of methylation (CG/CHG/CHH) within the B fragment were slightly higher in continuously flowering Purple branch than in OF Plena at all three time points (Fig. 5d), while it was higher only in April within the A fragment in continuously flowering Purple branch (Fig. S2a and S2c). Of the three types of methylation, CG and CHG methylation were most common (Figs. 5c and S2b). The methylation intensity within the promoter was negatively associated with the corresponding level of the *KSN* transcript presented in Fig. 3. Thus, hypermethylation may have partially contributed to the lower expression of *KSN* in Purple branch.

### Histone methylation of the *KSN* locus in Purple branch is modified

**Fig. 6:**
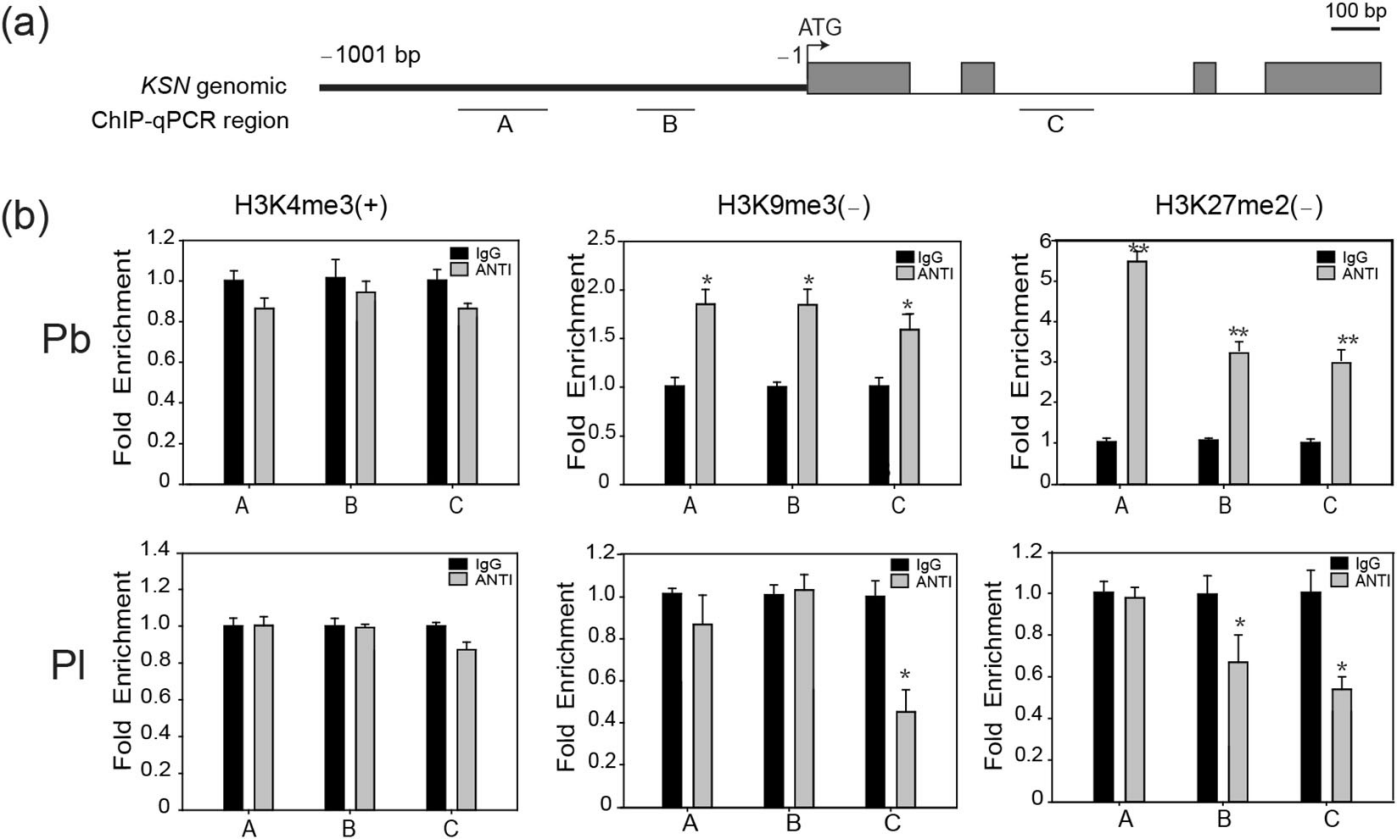
Histone methylation of the *KSN* locus in Purple branch and Plena. **a** Schematic representation showing ChIP-qPCR regions A (–638 to –496 bp), B (–387 to –240 bp) and C (494 to 595 bp), marked by black bars below the *KSN* genomic diagram. **b** Enrichment of H3K4me3, H3K9me3 and H3K27me2 in different regions of *KSN* from Purple branch (Pb) and Plena (Pl). IgG was used as a negative control, and ANTI indicates the corresponding antibodies. The error bars indicate the standard deviations (*n* = 3). The asterisks above the bars indicate significant differences, as determined by KS tests (*P < 0.05; **P < 0.01)

To investigate how histone modification regulates *KSN* expression, we collected shoot apices from field-grown Purple branch and Plena and analyzed *KSN* expression and the chromatin environment at the *KSN* locus in continuously flowering Purple branch and OF Plena via ChIP using H3K4me3-specific antibodies (to determine active loci) and H3K9me3- and H3K27me2-specific antibodies (to determine silent loci). We designed specific primers throughout the promoter and the second intron regions (Fig. 6a and Table S1) and performed ChIP-qPCR. *KSN* expression was obviously lower in Purple branch than in Plena (Fig. S3), which is in line with the previous results shown in Fig. 3. The histone methylation marks H3K9me3 and H3K27me2 increased in continuously flowering Purple branch but remained unchanged or decreased in OF Plena. The H3K4me3 mark decreased in continuously flowering Purple branch but remained unchanged in OF Plena (Fig. 6b). Thus, the enhanced histone methylation at H3K27 and H3K9 and the reduced methylation at H3K4 may play important roles in maintaining the lower expression of the normal *KSN* allele in Purple branch, leading to its CF behavior.

### *KSN* expression increases upon silencing *MET1*, *CMT3*, and *SUVR5*

In Arabidopsis, DNA methylation is mainly performed by members of the C5-MTase families. MET1, which belongs to the METHYLTRANSFERASE (MET) family, maintains CG methylation. CMT3, which belongs to the CHROMOMETHYLTRANSFERASE (CMT) family, maintains CHG methylation^33^. The *cmt3* mutant shows a near-complete loss of CpXpG methylation, and *met1* shows a marked reduction in CpG methylation; both types of methylation in turn cause gene silencing^34,35^. Histone modifications affect various changes in chromatin structure, leading to the promotion or suppression of gene expression^36^. *JMJ12* (*REF6*), which belongs to the KDM4/JHDM3 group of the JmjC family^37^, encodes a histone H3 lysine 27 demethylase^38^, and silencing of *RcJMJ12* induced late flowering in *Rosa chinensis*^39^. The SET family gene *SUVR5* regulates flowering time through H3K9me2 and H3K27me3 and is independent of the vernalization pathway^40–42^.

**Fig. 7:**
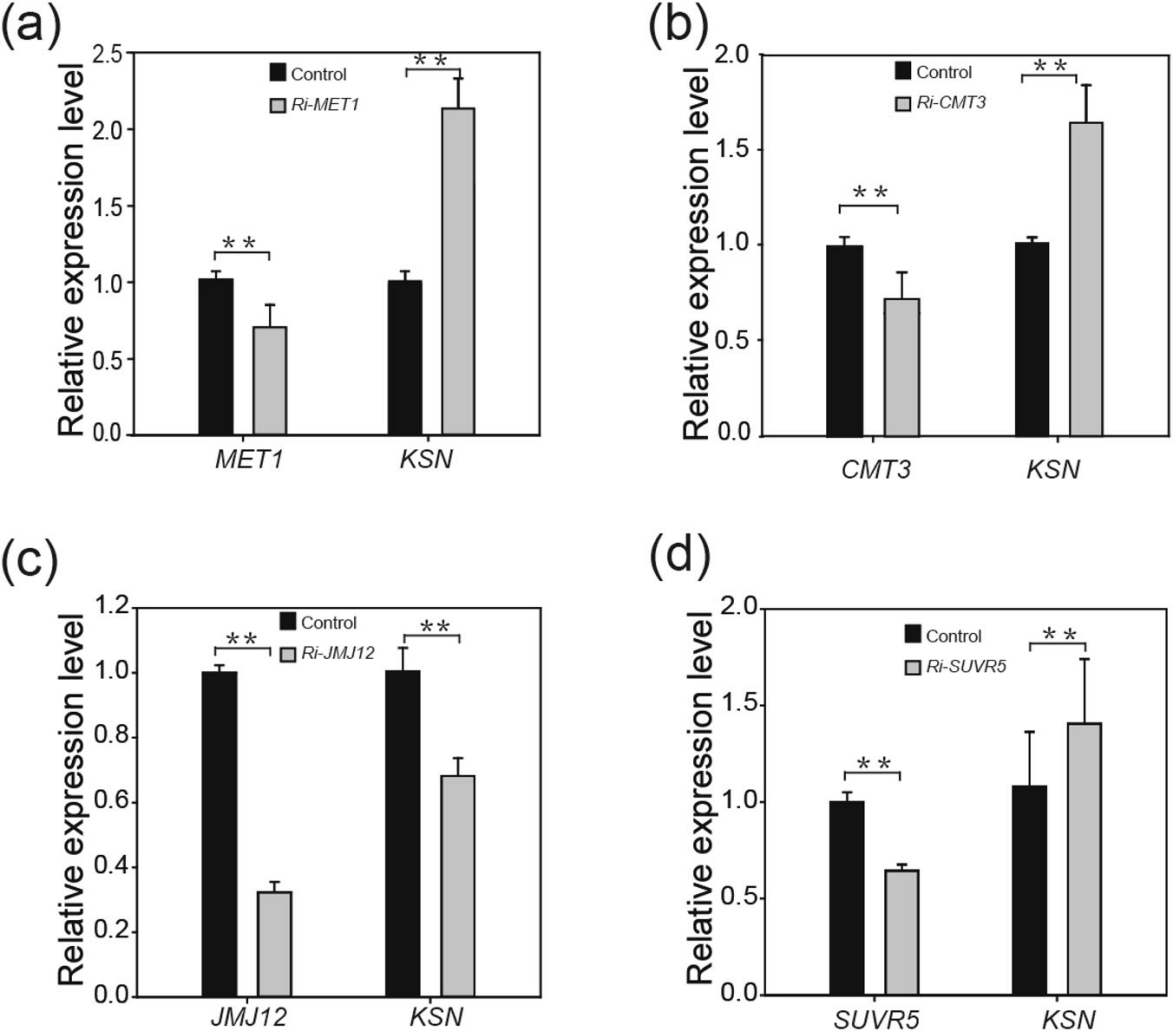
Silencing *MET1*, *CMT3*, and *SUVR5* increases *KSN* expression in Purple branch. **a**, **b** Expression levels of *MET1*, *CMT3*, and *KSN* in Purple branch seedlings of *Ri-MET1* and *Ri-CMT3* lines. **c**, **d** Expression levels of *JMJ12*, *SUVR5*, and *KSN* in Purple branch seedlings of *Ri-JMJ12* and *Ri-SUVR5* lines. Gene expression was measured via qRT-PCR for three biological replicates, with three technical repeats each. The *RcGADPH* gene was used as internal reference. The asterisks above the bars indicate significant differences, as determined by KS tests (**P < 0.01)

To examine the epigenetic regulation of *KSN* expression in Purple branch, we cloned the homologs of Arabidopsis *MET1*, *CMT3*, *JMJ12*, and *SUVR5* from continuously flowering Purple branch and constructed phylogenetic trees together with their homologs in Arabidopsis to verify their correctness before submitting the sequences to the NCBI database (Fig. S4). We then designed primers (listed in Supplemental Table S1) specific to their conserved regions and silenced the genes in young rose shoots using a transient transformation system described previously^28^. The subsequent qRT-PCR results showed that silencing *MET1*, *CMT3*, *JMJ12*, and *SUVR5* significantly altered the DNA methylation/histone acetylation status at the *KSN* locus (Figs. S5 and S6), which was followed by a significant increase in *KSN* transcription in *Ri-MET1*, *Ri-CMT3* and *Ri-SUVR5* seedlings compared with a decrease in *KSN* expression in *Ri-JMJ12* seedlings of continuously flowering Purple branch (Fig. 7). Thus, DNA methylation and histone methylation are potentially partially responsible for the repression of *KSN* in Purple branch.

## Discussion

Previous studies have genotyped *KSN* in a wide range of rose cultivars and established that some Damask-related roses (e.g., Damask, Moss, Hybrid musk, and Bourbon roses) and Asian roses (e.g., Hybrid rugosa and Hybrid bracteate roses) were CF types but contained no mutated alleles of *KSN* from *R. chinensis*^12^. Similarly, it was also found that continuously flowering *R. rugosa* Hamanasu from Japan had only a wild *KSN* allele without any insertion or obvious mutations^11^. Therefore, the CF behavior of rose plants may not be simply explained by the mutation or insertion of the *KSN* gene. Interestingly, the present results clearly showed that the *KSN* allele with the Copia insertion from *R. chinensis* was substantial in *R. rugosa* Purple branch (Figs. 2 and 3), which is heterozygous (*KSN-Copia*/*KSN-Wt-Pb*) at the *KSN* locus. The wild-type *KSN* allele was proven to be functional via heterologous expression in Arabidopsis (Fig. 4). This discrepancy may be caused by different experimental materials or methods, as there are different cultivars and varieties of *R. rugosa* from different origins. Additionally, the *KSN* allele carrying the 9 kb insertion was not easily amplified with the normal DNA polymerase and general PCR protocol.

Next, *KSN* expression was suppressed during the floral initiation period in early spring (April) and dramatically upregulated in the shoot apices after flower initiation in all three species, although *KSN* expression was still much lower in continuously flowering Purple branch than OF Plena and *R. davurica* in September (Fig. 3b). In contrast, expression of the floral activator *FT* significantly increased in April in all three species, decreased in OF Plena and *R. davurica* but continued climbing in continuously flowering Purple branch (Fig. 3c). Consequently, continuously flowering Purple branch exhibited high expression of both *FT* and *KSN* in September. Plant flowering is precisely determined by the equilibrium between floral promoters and repressors. FT, a member of the phosphatidylethanolamine-binding protein (PEBP) family, is a key florigen that integrates the internal and external signals for floral transition^43,44^. In the shoot apical meristem (SAM), FT interacts with FD to activate floral identity genes such as *AP1* and *LFY* and initiates floral bud formation^45,46^. Like the flowering promoter FT, TFL1 is also classified as a PEBP member^47^ but with a function opposite that of FT, acting as a floral repressor. TFL1 interacts with FD to form a TFL1-FD complex, opposing the function of the FT-FD complex in regulating downstream gene expression^48^. Thus, the floral activator FT may antagonize or suppress the repressor KSN in continuously flowering Purple branch, enabling its flowering in autumn.

Nevertheless, the overall expression of *KSN* was clearly linked to the alternation of vegetative/reproductive stages of rose plants in the present paper and in six other nonrecurrent flowering species and nine recurrent flowering cultivars^49^. Furthermore, the expression level of *KSN* in continuously flowering Purple branch was obviously lower than that in OF Plena and *R. davurica* at all three time points, which was convincingly associated with the CF trait of Purple branch. Although other factors may be involved in controlling the CF trait, the roles of reduced *KSN* expression should be predominant and can be mainly attributed to the decreasing dose associated with the heterozygosity of *KSN* loci in Purple branch (Figs. 2 and 3).

All plants have various transposons (TEs) that can disrupt local gene structure, affect the expression of nearby genes, and induce chromosomal instability. Most TEs are silenced and immobilized under normal conditions. DNA methylation and histone methylation are reversible epigenetic modifications that control transposon activity^50–52^. The 9 kb Copia-like retrotransposon insertion in *R. chinensis* Old blush not only blocks transcription of the host *KSN* allele but also results in a large rearrangement at the CF locus, leading to the complete deletion of the other *KSN* allele^11^. Therefore, the diploid Old blush is hemizygous: *RoKSN*^*Copia*^/*RoKSN*^*null*^. This allowed us to test the DNA and histone methylation status of the *KSN* locus. We observed hypermethylation of the *KSN* promoter and enhanced methylation levels of H3K9 and H3K27 at the *KSN* locus in continuously flowering Purple branch compared with OF varieties without Copia insertions (Figs. 5 and 6). These results implied that the presence of Copia-like retrotransposon insertion may potentially affect the epigenetic status of the *KSN* locus in continuously flowering Purple branch. Further silencing the DNA methyltransferase genes *MET1* and *CMT3* and histone methyltransferase gene *SUVR5* in young shoots significantly increased the expression of *KSN* in the Purple branch (Fig. 7), highlighting the importance of epigenetic modification in *KSN* expression. Taken together, these data suggested that the reduction in *KSN* expression may be associated with epigenetic modifications, except for the halved dose in continuously flowering Purple branch.

In conclusion, the *R. rugosa* Purple branch was found to be heterozygous at *KSN*, and its CF trait was associated with the lower expression of *KSN*. The reduction in *KSN* was caused by the halved dose and was also associated with hypermethylation of the promoter region and histone modification at the *KSN* locus following the 9 kb Copia insertion in the other allele. This suggests a novel mechanism for the production of the CF habit in rose plants.

## Supplementary information

Figure S6

Table S1

Figure S1

Figure S2

Figure S3

Figure S4

Figure S5
