# Supplementary figures and images for: KSN heterozygosity is associated with continuous flowering of Rosa rugosa Purple branch
Source: Hortic Res. 2021 Feb 1;8:26. doi: 10.1038/s41438-021-00464-8 (PMC7848002; doi:10.1038/s41438-021-00464-8)

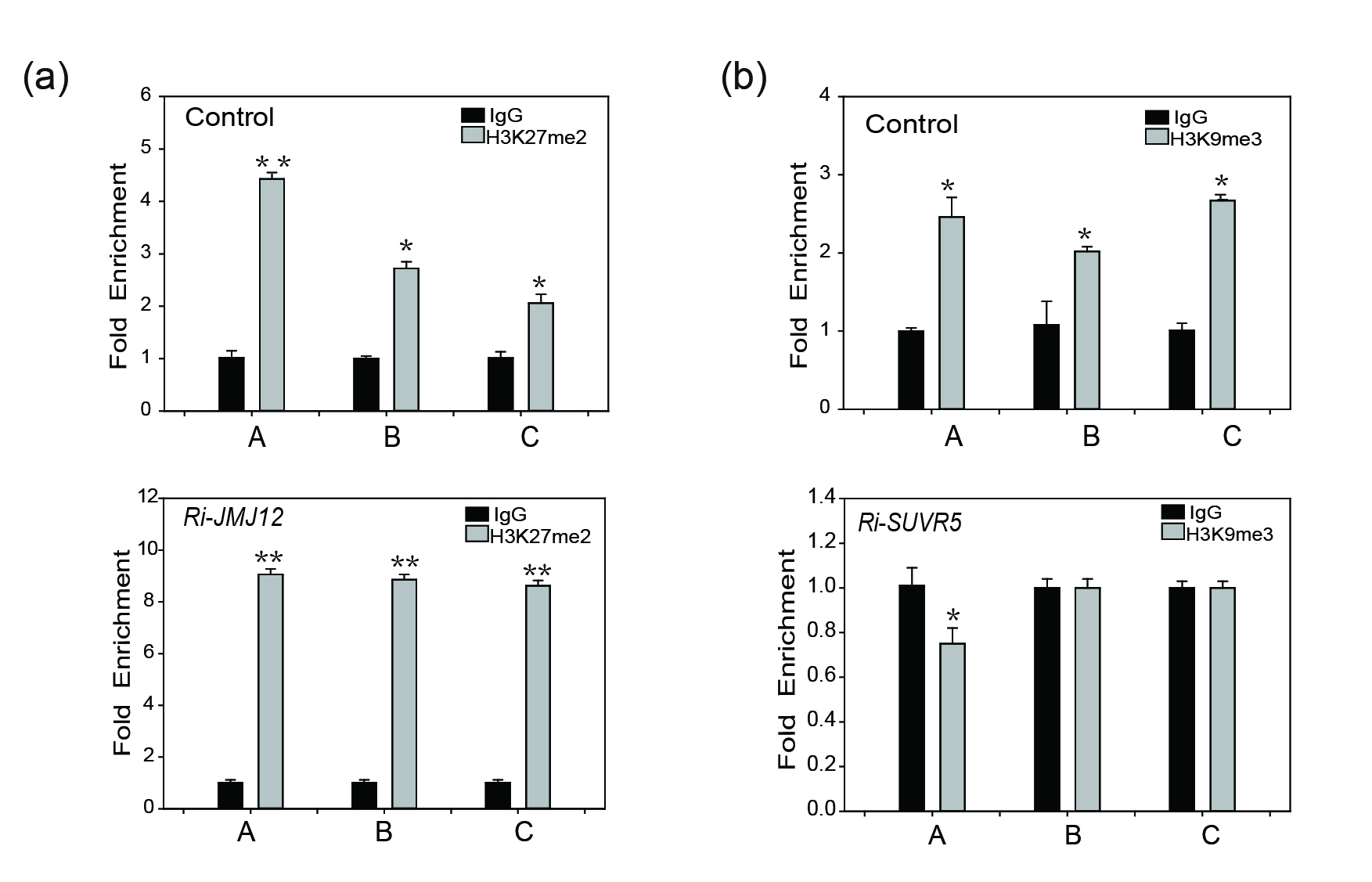

Supplement: Supplementary file 1 — Figure S6 [file 41438_2021_464_MOESM1_ESM.jpg]

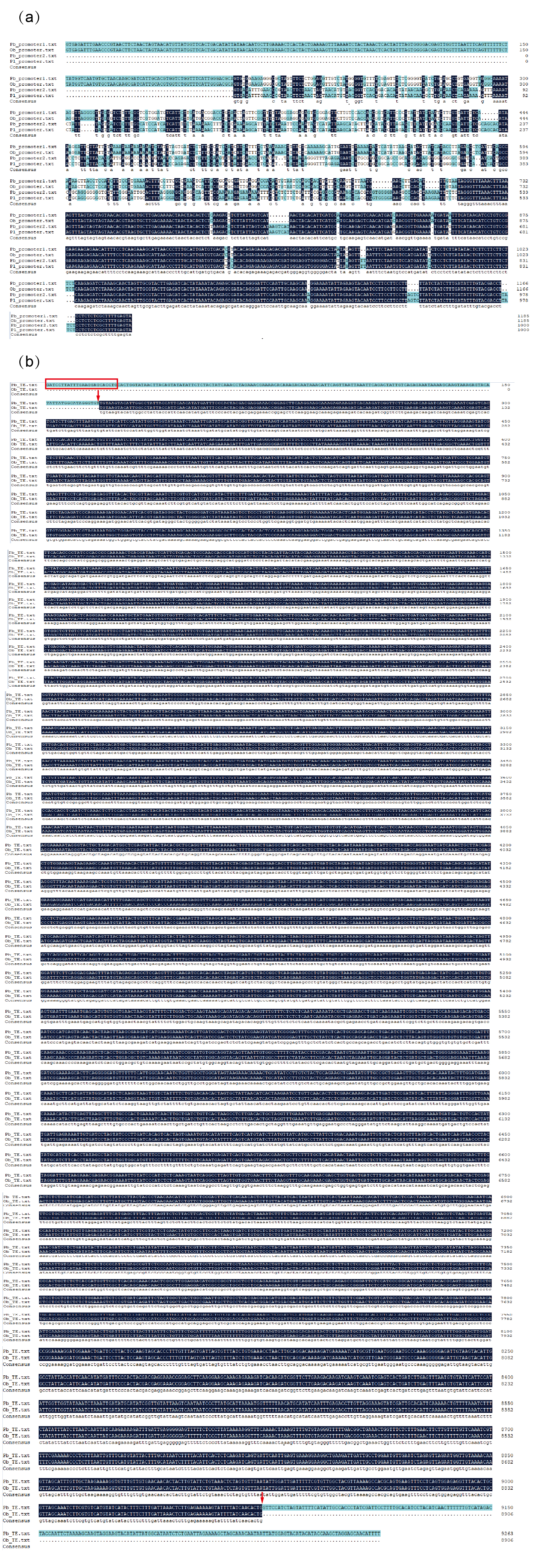

Supplement: Supplementary file 3 — Figure S1 [file 41438_2021_464_MOESM3_ESM.jpg]

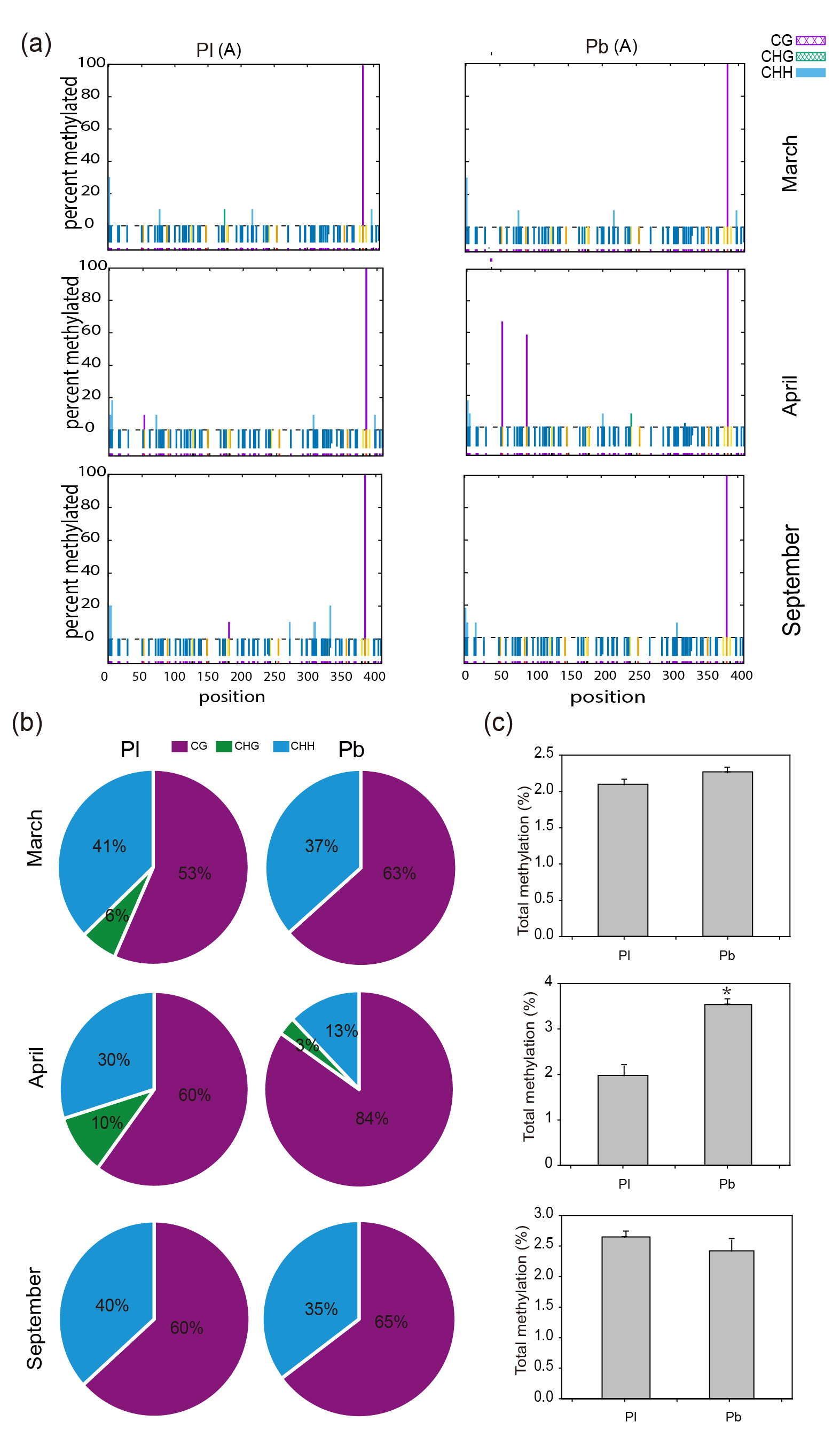

Supplement: Supplementary file 4 — Figure S2 [file 41438_2021_464_MOESM4_ESM.jpg]

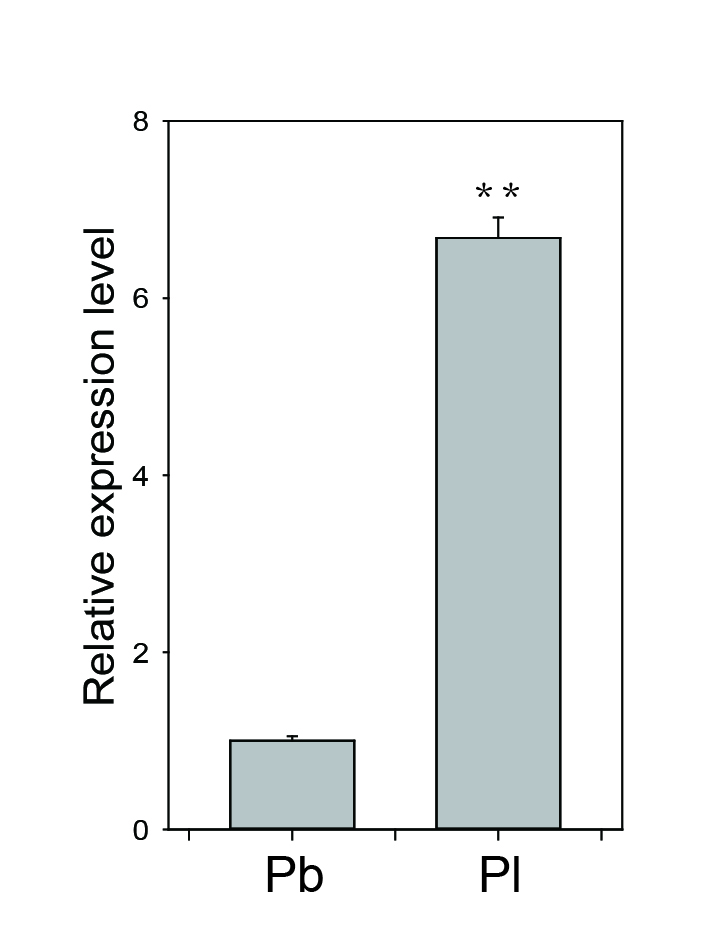

Supplement: Supplementary file 5 — Figure S3 [file 41438_2021_464_MOESM5_ESM.jpg]

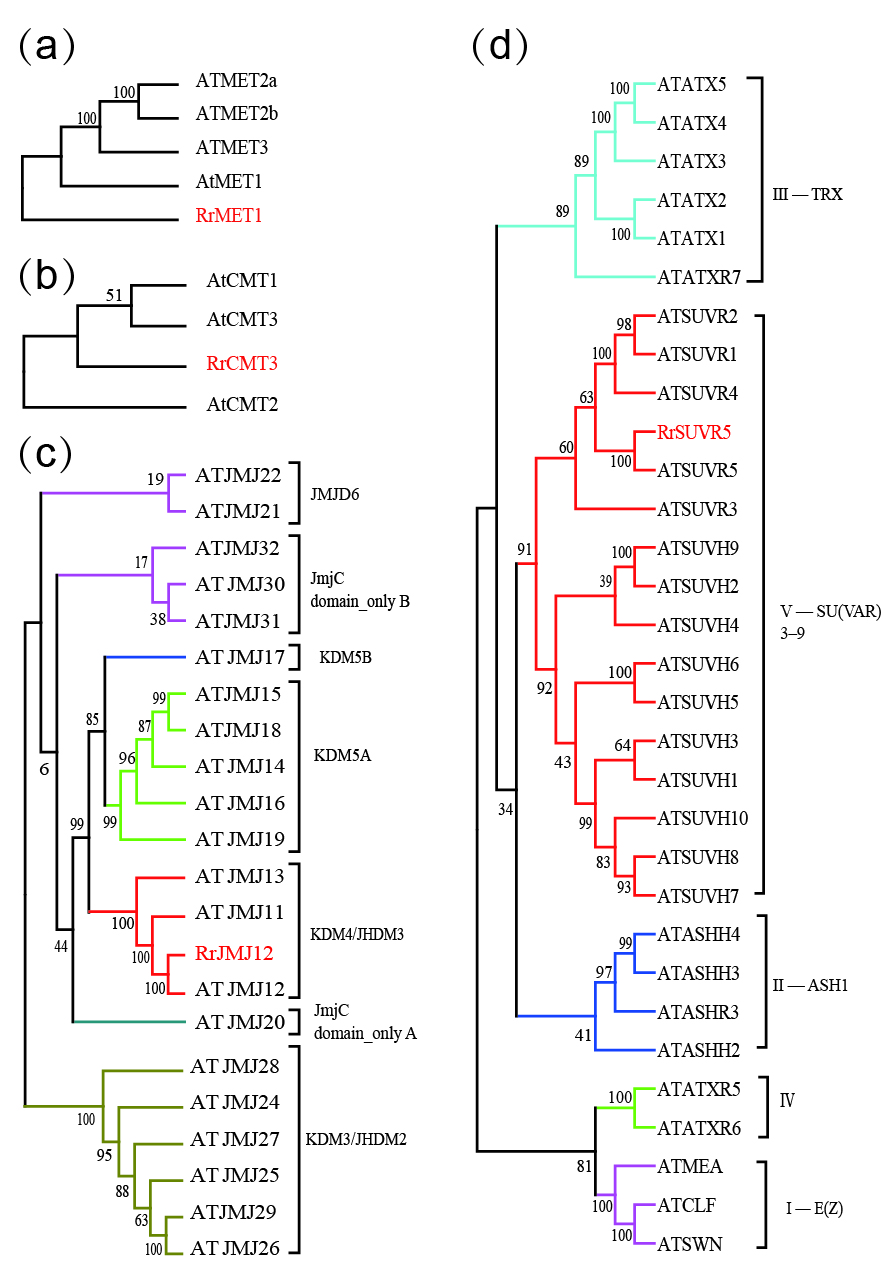

Supplement: Supplementary file 6 — Figure S4 [file 41438_2021_464_MOESM6_ESM.jpg]

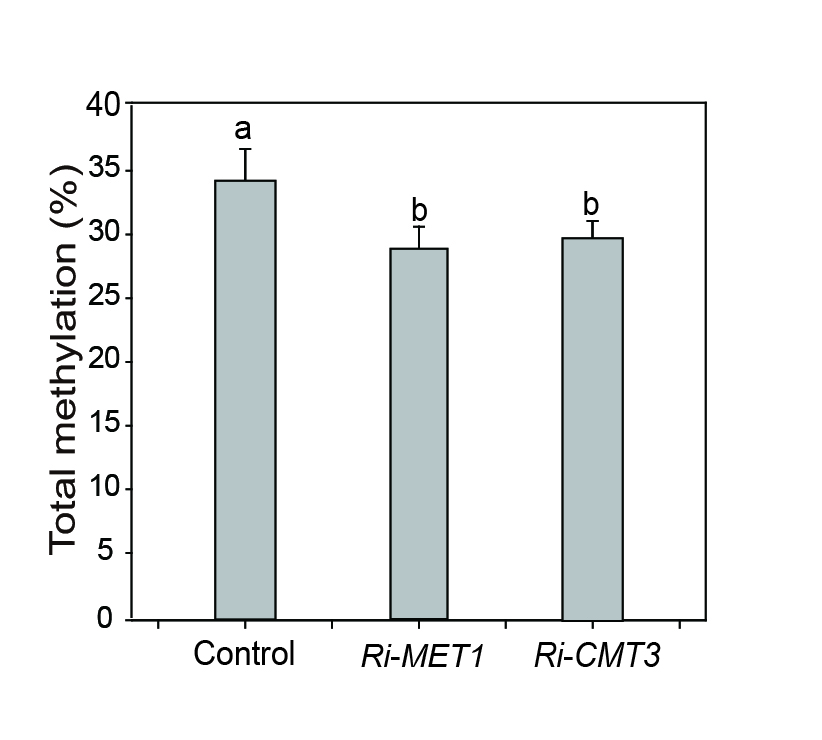

Supplement: Supplementary file 7 — Figure S5 [file 41438_2021_464_MOESM7_ESM.jpg]
