# Supplementary material for: KSN heterozygosity is associated with continuous flowering of Rosa rugosa Purple branch
Source: Hortic Res. 2021 Feb 1;8:26. doi: 10.1038/s41438-021-00464-8 (PMC7848002; doi:10.1038/s41438-021-00464-8)
Supplement: Supplementary file 2 — Table S1 [file 41438_2021_464_MOESM2_ESM.docx]

**Table S1 List of primers used in this paper**

| **Gene** | **Primers** | **Note** |
| --- | --- | --- |
| *pKSN* | F1- GTGAGATTTGAACCCGTAACTTC  R1- GTA CTCAAAAGCGAGAGAGGAGG | promoter cloning |
| *KSN* | F2- ATGGCAAGAATTTCGGAACCTTTAG  R2- GCGTCTTCTTGCTGCCGTTTCT | gene cloning |
| *KSN-qRT* | F-GATGACAGACCCAGATGTTCC  R- GATTCCTATGTTTGGCCTTGC | q-PCR |
| *pKSN* | A-F- TTAAATTTATTATTTAGTGGGGACGA  A-R- AAACCCTAAAAAAATTCTTAAATAAA  B-F- GTATTTTAGGGAGTAGGGGTAGT  B-R- ACTTACAATTAAAATCCCTATATCG | methylation-specific PCR (MSP) |
| *gKSN* | A-F- GGATCAATCACTGCCCAGCA  A-R- TTCTTAGGTGGGCGTTTCCC  B-F-TCATGCTGCAAGAGTCAACA  B-R-CCACCTCCCATCGTCTCTTT  C-F-AGACTATTGTCAGAGAAATAAAAGCAAG  C-R-GTTGATGTAGGATGTGAAAATGAATTG | ChIP-qPCR |
| *CMT3* | 1. ATGGAAGTGCTCCCTTGTTCGAGCGT   R-ATAGAGCCGGCCTCGGCATCTG | gene cloning |
| *CMT3-RNAi* | F-CTCACTCCGTTCTTTTCTCCGCTATC  R-GTCATCATATAGATCGTAAACAATCCC | RNAi cloning |
| *CMT3-qRT* | F-TTGCAGTTCCCGTTTCAAGG  R-GAATCTGCTCCGGAGGGTAA | q-PCR(RNAi) |
| *MET1* | F-ATGGGATCCGCCGCAGGATTAGAAC  R-ATCTTGTGAAGAAGACTTCTTCTTGCT | gene cloning |
| *MET1-RNAi* | F-AAGGGCCTGTTTGGAAGGTTAGACT  R-CGGGCCTATACGCTAGTGATAGTTGAG | RNAi cloning |
| *MET1-qRT* | F-AGAAGGTCTTGCTGTACGCT  R-ACCAGCTTCCACCATCTCAA | q-PCR(RNAi) |
| *JMJ12* | F-ATGGCAGCTTCGGAGCAGCCCC  R-TCGCGATCGCCCCTTACCTTTCTTTACG | gene cloning |
| *JMJ12-RNAi* | F-TTCAGCTGGTTTGCTTGGCA  R-TATTGCGCACAGGCGTTCTT | RNAi cloning |
| *JMJ12-qRT* | F-TCGGACTGAGCACATTAGGG  R-CTGACACAAACCGGAAGGTC | q-PCR(RNAi) |
| *SUVR5* | F-ATGGAAGTGCTCCCTTGTTCGA  R-ATAGAGCCGGCCTCGGCATCTGG | gene cloning |
| *SUVR5-RNAi* | F-CCCTTCTTTAAAGAGAGGTGGGAGGCA  R-ATGCGAACCACTAGATGACTTTTGGCT | RNAi cloning |
| *SUVR5-qRT* | F-GCCCAACAACCTGGACATTT  R-ACAAGCGTTGTGGCAAGATT | q-PCR(RNAi) |
| *FT-qRT* | F-AGCTTGTGAGTTGTGGGTCT  R-ATTGGGAACCGCCCAAGAAA | q-PCR |
